# Synopsis of proposals on fungal nomenclature: a review of the proposals concerning *Chapter F* of the *International Code of Nomenclature for algae, fungi, and plants* submitted to the XII International Mycological Congress, 2024

**DOI:** 10.1186/s43008-024-00151-y

**Published:** 2024-08-16

**Authors:** Tom W. May, Konstanze Bensch

**Affiliations:** 1 Royal Botanic Gardens Victoria, 100 Birdwood Ave, Melbourne, Victoria 3004 Australia; 2 Fungal Nomenclature Bureau, XII International Mycological Congress, Maastricht, The Netherlands; 3 Westerdijk Fungal Biodiversity Institute, Uppsalalaan 8, Utrecht, CT 3584 The Netherlands

**Keywords:** DNA sequence types, ICNafp, Governance, Guiding vote, Nomenclature, Protected names

## Abstract

A commentary is provided on the seven formally published proposals to modify the provisions of *Chapter F* of the *International Code of Nomenclature for algae, fungi, and plants* (ICNafp) that will be dealt with by the Fungal Nomenclature Session (FNS) of the 12th International Mycological Congress (IMC12) in August 2024. The proposals deal with: fungi whose morph-names have the same epithet; the listing of synonyms under entries for protected names in the *Code* Appendices; the processes of protection and rejection; the use of DNA sequences as nomenclatural types; the use of genomes as nomenclatural types; and the designation of fungi known only from DNA sequences. Information is also provided on the composition and role of the Fungal Nomenclature Bureau, the operation of the FNS and the pre-Congress Guiding vote.

## Introduction

*Chapter F* is the portion of the *International Code of Nomenclature for algae, fungi and plants* (ICNafp) that contains provisions that solely relate to organisms treated as fungi. The procedures for amending *Chapter F* are set out in the *Shenzhen ICNafp* (Turland et al. 2018). Proposals to amend *Chapter F* are dealt with by the Fungal Nomenclature Session (FNS) of an International Mycological Congress (IMC). A call for such proposals was published in 2020 (May 2020). A set of proposals to amend *Chapter F* to be considered at IMC12 was published recently (May & Hawksworth 2024). This Synopsis presents those proposals along with a commentary, following the established practice for proposals presented to an International Botanical Congress (IBC) (Turland and Wiersema 2024). This Synopsis has been prepared in our capacities as the appointed Secretary (TWM) and Deputy Secretary (KB) for the upcoming Fungal Nomenclature Session in Maastricht (*see below*).

### Fungal nomenclature session

The proposals discussed below will be formally considered at the Fungal Nomenclature Session (FNS) of the XII International Mycological Congress to be held on Thursday 15 August 2024 in Hall 6 of MECC Maastricht, The Netherlands. All persons registered for at least that day of the Congress are eligible to attend and vote in the FNS. Each person eligible to attend has one vote, and there are no institutional votes.

Procedures for the FNS are set out in Provision 8 of Division III of the *Shenzhen Code* (Turland et al. 2018). These procedures include: a qualified majority (60%) is required for accepting proposals and for referring items to the Editorial Committee; while a 50% majority is required for accepting an amendment to a proposal, for choosing between two alternative proposals, and for establishing and referring items to a Special-purpose Committee (SPC). Proposals solely concerning examples are automatically referred to the Editorial Committee. Changes to the wording of particular proposals may be moved as amendments during the FNS, either as a friendly amendment [when accepted by the original proposer(s)] or otherwise when introduced by an eligible attendee of the FNS and seconded by five other eligible attendees.

The FNS may authorize one or more Special-purpose Committees, with a specific mandate, to be appointed by the Nomenclature Committee for Fungi in consultation with the General Committee, that report back to the FNS of the next IMC. Examples of previous Special-purpose Committees are the Special Subcommittee on Governance of the *Code* with Respect to Fungi, appointed following the Melbourne IBC, that produced proposals to amend the *Code* that were ultimately adopted at the Shenzhen IBC (May 2016; Hawksworth et al. 2017) and the Special-purpose Committee on Names of Fungi with the Same Epithet that will report to IMC12 (Mitchell et al. 2024).

For the *Code*, an Editorial Committee is elected by the Nomenclature Section of an IBC, and that Committee finalizes the *Code* arising from that Congress. After the last IMC, a separate “San Juan *Chapter F*” was published (May et al. 2019) prepared by an ad hoc “Editorial Committee - *Chapter F*”. A formal proposal to amend Division III of the *Code* to formalise an “Editorial Committee for Fungi” has been submitted for consideration by the IBC Nomenclature Section (May et al. 2023). If that proposal is accepted, an Editorial Committee for Fungi will be appointed at the IMC12 FNS.

Because the IBC Nomenclature Section will be meeting in July 2024 (in Madrid), shortly before the IMC, it is not expected that there will be a need for a separate publication of *Chapter F* resulting from any decisions of the IMC Nomenclature Session. Any revisions to *Chapter F* arising from the IMC12 FNS will be passed on to the Editorial Committee who prepares the Madrid *Code*, which is expected to appear in 2025.

### Fungal nomenclature bureau

The Fungal Nomenclature Bureau (FNB) of an International Mycological Congress is responsible for running the FNS and the pre-Congress Guiding vote. The FNB consists of the Chair, up to five Deputy Chairs, Secretary, Deputy Secretary, and Recorder. These roles in the FNB are the equivalent of the President, Vice-president, Rapporteur-général, Vice-rapporteur and Recorder in the Bureau of Nomenclature of an International Botanical Congress. The roles are approved by various combinations of the preceding International Mycological Congress (May et al. 2018), the Nomenclature Committee for Fungi (May & Lendemer 2023), the General Committee (Wilson 2024) and the organizing committee for the International Mycological Congress — see Division III Provision 8 of the *Code* for details. The officers of the FNB for IMC12 are: Amy Rossman (Corvallis, USA; Chair), Tom May (Melbourne, Australia; Secretary), Konstanze Bensch (Utrecht, The Netherlands; Deputy Secretary) and Jos Houbraken (Utrecht, The Netherlands; Recorder). David Hawksworth (United Kingdom) was appointed as Emeritus Deputy Chair at IMC11 and remains in that position.

The Deputy Secretaries will be appointed by the FNB closer to the Congress. Further details on the election and duties of members of the FNB can be found in Hawksworth et al. (2017). In his role as Rapporteur-général for the Madrid IBC, Nicholas Turland (Berlin, Germany) has kindly agreed to an invitation from the International Mycological Association to attend the Fungal Nomenclature Session in Maastricht as an observer.

As Secretary and Deputy-Secretary of the FNB, we provide here a Synopsis of the proposals, as background for the pre-Congress guiding vote and for the deliberation of the FNS of IMC12. The Synopsis is not intended as a vehicle for the personal opinions of the secretaries, but rather is an opportunity to draw together all the proposals and examine technical aspects such as clarity of wording, ramifications for other articles, and unexpected consequences, as well as indicate opinions of relevant international committees.

The seven formal proposals to amend *Chapter F* have been submitted for publication in *IMA Fungus* (May & Hawksworth 2024). In case the article with the bundle of proposals does not appear on-line prior to the commencement of the Guiding vote, a pre-publication version is available on the IMA website at: <https://www.ima-mycology.org/index.php/formal-proposals>.

### Guiding vote

A pre-Congress Guiding vote will take place subsequent to the publication of this Synopsis, until 2 August 2024 as a non-binding but nevertheless indicative assessment for the FNS on the published proposals. Any proposal that has a "No" vote that is equal to or greater than 75 % in the Guiding vote is automatically rejected by the FNS, unless a proposal to discuss it is moved by a registered attendee of the FNS and seconded by five other registered attendees.

Details on the operation of the Guiding vote for the San Juan IMC were provided by May & Miller (2018). Participation in the Guiding vote is open to authors of proposals, members of the Nomenclature Committee for Fungi, and members of a range of organizations as set out in Division III of the *Code*, specifically the International Mycological Association and its Member Mycological Organizations along with additional organisation as approved by the FNB.

Organisations whose members are eligible to participate in the Guiding vote are listed on the IMA website <http://www.ima-mycology.org/nomenclature/guiding-vote>. Results of the Guiding vote will be available on the IMA website prior to the IMC at the same link.

It is recommended to read this Synopsis in parallel with the original proposals (May & Hawksworth 2024) before completing the Guiding vote. A pre-publication version of the bundled set of proposals is available on the IMA website at: <https://www.ima-mycology.org/index.php/formal-proposals>.

Options on the Guiding vote, for each proposal will be: **No**, **Yes**, **Special-purpose Committee**, **Editorial Committee**, and **Abstain**. A “Yes” vote only implies approval in principle of the proposal, not necessarily of its exact wording. An “Editorial Committee” vote (unless otherwise indicated) instructs the *ad hoc* Editorial Committee - *Chapter F* to consider inclusion in the *Code* of material in the proposal, but does not require it to do so.

### Opinions of committees

The proposals to amend *Chapter F* of the *Code* were submitted to the Nomenclature Committee for Fungi (NCF) and the International Commission on the Taxonomy of Fungi (ICTF) for their opinion, with the options Yes – No – Abstain. In the NCF 14 out of the 15 members voted and in the ICTF, 13 of the 20 members voted. Percentages are of the members voting. A given committee is stated to support a proposal when the "Yes" vote is 50 % or more.

### Proposals to conserve, protect or reject and requests for binding decisions

It is important to note that proposals to conserve, protect or reject names or to suppress works and requests for binding decisions (such as on confusability of names) are submitted to the General Committee (GC) for examination by the relevant Specialist Nomenclature Committee, which for fungi is the Nomenclature Committee for Fungi (NCF). The means of submitting proposals and requests to the GC is *via* publication in the journal *Taxon*, except for lists for protection or rejection, prepared by working groups established under Art. F.2 or F.7, which are published in *IMA Fungus*. The most recent reports of the NCF were published in *Taxon* (May 2024a, 2024b; May & Lendemer 2023) and reports of the GC appear in *Taxon*. **The proposals and requests dealt with by the NCF (and ultimately the GC) are not part of the business of the FNS**.

## Proposals to amend *Chapter F* of the *code*

This Synopsis repeats the exact wording of the proposed changes to *Chapter F* (May & Hawksworth 2024). The authors specific to the proposal are included in square brackets. Numbering of articles and recommendations and the quoted text follows the *Shenzhen ICNafp* (Turland et al. 2018) and the *San Juan Chapter F* (May et al. 2018). When existing articles are quoted, new text is in **bold**, deleted text is in strikethrough. Entirely new Articles, Notes and Recommendations are all in **bold**. Proposals below relate to Articles (Art.), Recommendations (Rec.) and Examples (Ex.) of the *Code*. The sequence of presentation of proposals below follows the numbering sequence of existing Articles in *Chapter F*, with material related to two newly proposed sections of *Chapter F* presented at the end as Sections X and Y. Should one or more proposals be accepted, material in *Chapter F* will be renumbered as appropriate.

### Art. F.2.1 – concerning the listing of synonyms in entries for protected names in the Appendices to the *Code* and clarifying the process of protection

*Prop. F-003* [May] Amend Art. F.2.1 as follows and add a new Note (new text in bold, deleted text in strikethrough)

“*F.2.1.* In the interest of nomenclatural stability, for organisms treated as fungi, lists of names proposed for protection may be submitted to the General Committee, which will refer them to the Nomenclature Committee for Fungi (see Div. III Prov. 2.2, 7.9, and 7.10) for examination by subcommittees **may be** established by **the Nomenclature** Committee **for Fungi (see Div. III Prov. 7.2)** in consultation with the General Committee and appropriate international bodies **for the purpose of preparing lists of names proposed for protection and/or rejection (see Art. F.7.1) for submission to the General Committee (see Div. III Prov. 2.2, 7.9, and 7.10)**. Protected names on these lists, which become part of the Appendices of the *Code* (see App. IIA, III, and IV) once reviewed and approved by the Nomenclature Committee for Fungi and the General Committee (see Art. 14.15 and Rec. 14A.1), are to be listed with their types and are treated as conserved against any competing listed or unlisted synonyms or homonyms (including sanctioned names), although conservation under Art. 14 overrides this protection. The lists of protected names remain open for revision through the procedures described in this Article (see also Art. F.7.1).”

*“Note 1*. Names in lists of names proposed for protection may be proposed with or without the listing of synonyms.”

***Secretaries' comments*** There are two aspects to this proposal. The first is to alter the wording to more clearly explain the process of establishing subcommittees [which in practice have been joint NCF/ICTF (International Commission on the Taxonomy of Fungi) Working Groups] and preparing, reviewing and approving the lists. The mechanics of the process are not being altered but the suggested amendments provide a useful clarification of the existing processes when lists of names are protected under Art. F.2.1.

The second aspect of the proposal is to remove the listing of synonyms from protected names that are entered into the *Code* Appendices. This change is sensible because the list of synonyms for any given protected name is not static but may change over time (due to changes in taxonomy). It is not the function of the Code *Appendices* to provide a full synonymy for each name, but rather to record those names for which formal nomenclatural action (such as conservation, rejection or protection) has been carried out. Synonyms can be determined from taxonomic publications and databases. The proposed change to remove the listing of synonyms for protected names will reduce the workload when relevant Appendices are prepared. We recommend that, should this proposal be successful, the synonyms of protected names currently listed in the *Code* Appendices be removed.

The NCF strongly supports Prop. F-003, with a 100 % Yes vote (14 – 0 – 0), as does the ICTF, with a 92% Yes vote (12 – 0 – 1).

### Art. F.5 Note 3 - clarifying that a proposal to conserve a name with a conserved type does not require citation of a typification identifier

*Prop. F-002* [May, Parra, Thines & Lendemer] Amend Article F.5 Note 3

“F.5.4. For purposes of priority (Art. 9.19, 9.20, and 10.5), designation of a type, on or after 1 January 2019, of the name of an organism treated as a fungus under this Code (Pre. 8), is achieved only if an identifier issued for the type designation by a recognized repository (Art. F.5.3) is cited.

Note 3. Art. F.5.4 applies only to the designation of lectotypes (and their equivalents under Art. 10), neotypes, and epitypes; it does not apply to the designation of a holotype when publishing the name of a new taxon, for which see Art. F.5.2**, nor does it apply to proposing a conserved type when publishing a proposal to conserve a name (Art. 14.9)**.”

***Secretaries' comments*** The proposal provides a useful clarification that a typification identifier is not needed when publishing a conservation proposal with a conserved type. It does not introduce any new procedure but merely points out something that is current practice.

The NCF strongly supports Prop. F-002, with a 100 % Yes vote (14 – 0 – 0), as does the ICTF, with a 92% Yes vote (12 – 0 – 1).

### Art. F.5.5 – concerning the designation of fungal organisms only known from DNA sequence data

*Prop. F-007* [Hawksworth, Kirk & Lücking] Insert a new Recommendation and Example

**“Recommendation F.5n. Identifiers can be issued by a recognized repository for sequence-based designations where there is no specimen or illustration available to serve as a nomenclatural type, but when released after effective publication such designations should have “nom. seq.” (nomen sequentium) appended to indicate that the designations are not validly published.**

***Ex. X. The designation Hawksworthiomyces sequentia de Beer & al. (in Fungal Biology 120: 1332. 2016) was assigned the identifier MB815690, but as it lacks a Code-compliant type it is to be referred to as H. sequentia de Beer & al. nom. seq. or H. sequentia nom. seq., but not as H. sequentia. The designation can become available for use upon valid publication (Art. 32-45) with a Code-compliant type.”***

***Secretaries' comments*** This proposal introduces a recommendation to be considered for inclusion in *Chapter F* should any proposals related to allow DNA sequences as types for fungi (see Prop. F-005 and F-006 below) not be successful.

We note that there is nothing to prevent authors of names based on DNA sequences alone (that are consequently currently invalid) from utilising informal devices such as “nom. seq.” but that standardising such devices has merit.

To date, very few names (strictly designations, as names based solely on DNA sequences are not valid) fall under this proposal — less than ten that we are aware of. At large scale, the issuing of identifiers for numerous non-valid names may be problematic for repository curators.

The proposal argues that unless the interim solution of “nom. seq.” is adopted there is a danger that some parallel breakaway system could develop outside of the *Code*. We note that UNITE (Abarenkov et al. 2023) already assigns unique and versioned digital identifiers to taxon concepts based on DNA sequences alone, such as the “species hypotheses” (SHs) at species level. While UNITE does not use binomials for undescribed species known only from DNA sequences, it provides a robust mechanism of generating identifiers at scale in terms of coping with large numbers of SHs based on DNA sequences alone. UNITE is not in competition with *Code*-compliant names but can be considered to provide a complementary system to the set of formally registered binomial names.

Within the NCF there were mixed opinions on Prop. F-007 (5 – 9 – 0), with a 64% No vote. Within the ICTF there was support for the proposal, with a 54% Yes vote (7 – 4 – 2).

### Art. F.7.1 - clarifying the processes of rejection

*Prop. F-004* [May] Amend Art. F.7.1

“*F.7.1.* In the interest of nomenclatural stability, for organisms treated as fungi, lists of names proposed for rejection may be submitted to the General Committee, which will refer them to the Nomenclature Committee for Fungi (see Div. III Prov. 2.2, 7.9, and 7.10) for examination by subcommittees **may be** established by that **the Nomenclature** Committee **for Fungi (see Div. III Prov. 7.2)** in consultation with the General Committee and appropriate international bodies **for the purpose of preparing lists of names proposed for protection (see Art. F.2.1) and/or rejection for submission to the General Committee (see Div. III Prov. 2.2, 7.9, and 7.10)**. **Rejected n**ames on these lists, which become part of the Appendices of the *Code* once reviewed and approved by the Nomenclature Committee for Fungi and the General Committee (see Art. 56.3 and Rec. 56A.1), are to be treated as rejected under Art. 56.1, except that they may become eligible for use by conservation under Art. 14 (see also Art. F.2.1).”

***Secretaries' comments*** The proposed wording changes parallel those proposed for Art. F.2.1 above. There is no alteration to the application of the Article. The changes, as with those proposed for Art. F.2.1, provide a useful clarification of the existing processes when lists of names are rejected under Art. F.7.1.

The NCF strongly supports Prop. F-004, with a 100 % Yes vote (14 – 0 – 0), as does the ICTF, with a 92% Yes vote (12 – 0 – 1).

### Art. F.8 - enabling the same epithet to be retained for different morphs of the same fungus

*Prop. F-001* [Hawksworth, de Hoog, McNeill & Wingfield] Insert a new provision in Art. F.8

“F.8.2. If, prior to 1 January 2013, an author publishing a new species name for the morph of a fungus that had an earlier name typified by a different morph adopted the specific epithet of the name of the previously described morph, the newly published name is to be treated as a new combination and not the name of a new taxon with a different type. Designations such as “sp. nov.” and ascriptions excluding the earlier name are to be treated as formal errors requiring correction.”

***Secretaries' comments*** This proposal addresses an issue that arose with the change in the Melbourne *Code* that removed dual nomenclature that allowed the separate naming of different morph states (asexual and sexual) of non-lichen-forming *Ascomycota* and *Basidiomycota*.

So-called “names with same epithets” exist where an earlier name applied to one morph state has a corresponding later name that applies to another morph, that uses the same epithet. Should there be heterotypic synonyms described between the publication of the two names with the same epithet, when the current name of the taxon is in a genus the name of which has been used for the later-described morph name, the earlier morph name cannot be transferred to the relevant genus (otherwise a homonym would be created). Thus, the epithet of the heterotypic synonym has to be taken up, potentially (although not in all cases) displacing an established name for a fungus of importance.

We note that Ex. 2 under Art. F.8 indicates that some later names with the same epithet that prior to 2012 were treated as new names (even though there were introduced as new combinations) are, under the current *Code*, treated again as new combinations. Therefore, this proposal deals specifically with cases where the later name with the same epithet was not introduced as a new combination.

The same proposal was made to the Nomenclature Section of IBC XIX in Shenzhen. It was not approved, but a Special-purpose Committee was set up to examine the issue, initially reporting to IBC XX and then, through action at the IMC11 Nomenclature Session (and consultation with the General Committee), reporting to IMC12 in Maastricht (May et al. 2018). The Special-purpose Committee on Names of Fungi with the Same Epithet has submitted its final report (Mitchell et al. 2024).

A question within the SPC concerned the number of cases to which this proposal would apply, should it be approved. Cases appear to number in the many hundreds, but the SPC found that it was not possible to document all cases, as links between morph states are not systematically recorded in the global nomenclatural databases. Nor was it possible to provide a list of all cases where it would be advantageous to apply Prop. F-001. Some specific cases are mentioned in Mitchell et al. (2024) and in the background to the proposal.

The members of the Special-purpose Committee were split on actions in relation to this proposal. As reported by Mitchell et al. (2024): “In the end, the Committee could not reach a consensus. Some members supported the proposed change as a common-sense fix to a problem created by an unfortunate historical practice, which was subsequently formalized. Other members favored employing already-existing methods for protection or conservation of these names (perhaps with additional streamlining), feeling the proposed change to be unnecessarily drastic for the scale of the problem.”

We note that under this proposal the original type of the second name (whatever morph, asexual or sexual) will cease to have any nomenclatural status. It simply becomes a specimen cited in connection with a new combination. It could only be used as an epitype if the original name was demonstrably ambiguous (Art. 9.9), and the original type of the second name possessed characters that would contribute to removing such ambiguity.

A technical aspect that should be considered is how to keep track of the nomenclatural action of changing the status of a new name to a new combination. Already, when nomenclatural novelties (such as new names and new combinations) and new typifications (such as lectotypifications) are published, it is mandatory for fungi that an identifier is cited (Art. F.5.1 and F.5.4). At least, it could be useful to include a recommendation that the “conversion” of a new name to a new combination, as allowed for in this proposal, be registered through citation of an identifier issued by a registered repository.

A majority (60% of five voting) of the Special-purpose Committee members were in favour of using a list of protected names to deal with the issue — with a proposed turnaround time for approval of less than a year. In relation to time frames, it is relevant that proposals to conserve, reject or protect names take effect “once approved by the General Committee after study by the specialist committee for the taxonomic group concerned” (Art 14.15). Over the last decade there have been long delays between the time of publication of proposals relevant to fungi and publication of the decisions of the Nomenclature Committee for Fungi. However, recent efforts by the NCF are reducing the backlog and the time between publication of NCF reports and publication of reports of the General Committee (GC) is also reducing. GC Report 30 (Wilson 2024) that appeared in April dealt with proposals in NCF Report 23 (May 2024a) that appeared in January.

A final vote on the issue within the Special-purpose Committee (seven members voting) was 3 for and 4 against the proposal (i.e. 57% No). Within the NCF there were also mixed opinions on Prop. F-001 (6 – 8 – 0), with a 57% No vote. However, there was support for the proposal within the ICTF, with a 69% Yes vote (9 – 3 – 1).

### Section X (new) - concerning DNA sequences as types

*Prop. F-005* [Hibbett, Nilsson, Groenewald, Hallen-Adams, Lendemer, Phukhamsakda, Rosling, Thines & May] Introduce a new Section X in *Chapter F* “DNA sequences as types”, with new articles and notes

“*F.X.1.* For organisms treated as fungi, on or after 1 January 2026, the holotype (Art. 9.1) may be a DNA sequence (see Art. F.X.2) if, and only if, preservation of a physical specimen or isolation and maintenance of a pure culture (preserved in a metabolically inactive state) is technically unfeasible.”

“*Note x.* For the purposes of Art. F.X.1, preservation is regarded as technically unfeasible if, and only if, physical specimens or pure cultures cannot reasonably be obtained using technologies available at the time of publication. Preservation is not considered unfeasible if a specimen or pure culture could not be obtained merely for reasons of inconvenience, lack of access or facilities, or if a specimen or culture was lost or otherwise not collected or isolated when it could have been.”

“*F.X.2*. For organisms treated as fungi, in order to be validly published (see also Art. 39.2) a name of a new taxon introduced with a DNA sequence as a holotype (Art. F.X.1) must be accompanied by both *(1)* citation of an identifier issued for the holotype sequence by a recognized online repository (see Art. F.X.5(a) and App. X) and (2) a diagnosis that compares informative portions of the holotype sequence against comparable sequences of inferred phylogenetic relatives. The citation in (1) and the specification in (2) must be in English.”

“*F.X.3.* For organisms treated as fungi that have a DNA sequence as a holotype (Art. F.X.1), an epitype (Art. 9.9) may be a DNA sequence. In order to effectively designate an epitype that is a DNA sequence, the identifier issued for the epitype sequence by a recognized online repository (Art. F.X.5(a) and App. X) must be cited, and *(b)* a diagnosis that compares informative portions of the epitype sequence against comparable sequences of inferred phylogenetic relatives must be provided.”

“*F.X.4.* In order to be validly published with a DNA sequence as type (see Art. F.X.1), in addition to meeting the requirements of Art. F.X.2 a name must *(a)* be published in an approved journal (see App. Y, Art. F.X.5(b)) and *(b)* be accompanied in the protologue by *(1)* a statement as to why it is believed that the taxon is new and unnamed, and *(2)* an explanation of why it was not feasible for a type specimen to be isolated, cultured, or otherwise prepared.”

“*F.X.5.* The Nomenclature Committee for Fungi in consultation with the General Committee, after seeking advice from relevant specialist committees and international societies, has the power to *(a)* appoint one or more localized or decentralized, open and accessible electronic repositories to issue the identifiers required by Art. F.X.2 and F.X.3 (see App. X), *(b)* ratify a list of approved journals for valid publication of names with DNA sequences as types (see App. Y), and *(c)* cancel or alter such appointments or ratifications at its discretion.”

“F.X.6. The responsibility of *(a)* maintaining a list of approved repositories for storing sequences and issuing sequence identifiers (Art. F.X.5(a)), and (*b*) maintaining a list of approved journals for valid publication of names with DNA sequences as types (Art. F.X.4(a) and F.X.5(b)) rests with the Nomenclature Committee for Fungi (Div. III Prov. 7.1(g)).”

***Secretaries' comments*** Much has been written on the pros and cons of introducing types that are DNA sequences (e.g. Thines et al. 2018; Zamora et al. 2018; Lücking et al. 2021; Nilsson et al. 2023).

Formal proposals to allow DNA sequences as types that were made to the Shenzhen IBC and to the San Juan IMC were not successful.

The topic has been the subject of two Special-purpose Committees – the first (considering the issue in relation to all organisms covered by the *Code*) reporting to the Madrid IBC and the second (considering the issue only in relation to fungi) reporting to the Maastricht IMC. The Madrid SPC has published a discussion paper (Thiele et al. 2023a) and a final report (Lehtonen & Thiele 2023) and two sets of proposals arose from their discussions, one of which treated DNA sequences as types (Thiele et al. 2023b). The discussions of the Maastricht SPC have resulted in two sets of proposals, under consideration here (F-005 and F-006). A report has not yet been published from the Maastricht SPC.

Prop. F-005 modifies the set of proposals made by Thiele et al. (2023b), to be considered at the Madrid IBC. The Thiele et al. (2023b) proposals are worded to apply to all organisms covered by the Code in circumstances where it is not feasible to preserve a type that is a specimen (or a culture stored metabolically inactive). The Thiele et al. (2023b) proposals had a very high “No” vote in the recent Guiding vote in relation to proposals for IBC XX (Turland et al. 2024) and will not be considered at IBC unless there is a proposal to reintroduce them (that has at least five seconders).

For those in support of DNA sequences as types, proposal F-005 offers a straightforward mechanism to do so. A DNA sequence that is lodged in an approved repository may be cited as the type specimen of a new species that is diagnosed on the characters of that sequence in comparison to “inferred phylogenetic relatives”. The proposal is an evolution from Prop. F-005 as put forward at the San Juan IMC, which allowed for species of fungi to be based on DNA sequences in any circumstances, whereas the current Prop. F-005 restricts DNA sequences as types to situations where it is not feasible to preserve a specimen (or metabolically inactive culture).

Nothing in the current *Code* prevents the publication of new species in self-published non-peer-reviewed publications. Consequently, there is a well-founded concern that a large number of new species based solely on DNA could be published by simply mining sequence repositories and creating phylogenetic trees (without necessarily having appropriate taxon sampling) and diagnoses in a semi-automated process. For this reason, this proposal includes a control on the taxonomic practice by mandating that new species based solely on DNA be published only in one or more specified journals that must be on a list of approved journals.

It is widely accepted that there is no single DNA marker that unambiguously separates all fungi at species level. In a circumstance where the DNA sequence chosen as a marker is inappropriate, the peer-review process of the specified journals is the control mechanism built into the proposal.

We note that for Prop. F-005 and F-006, the current requirements for citation of an identifier when introducing nomenclatural novelties (Art. F.5.1) would remain in force. Should either proposal be successful, cross references to Art. 5.1 should be added for clarity.

Prop. F-005 includes the option of epitypifying a name based on a DNA sequence as a type (the proposed Art. F.X.3). According to the proposal, only a name already based on a DNA sequence can be epitypified by a DNA sequence. There does not seem to be much practical use for epitypification of this nature as the only way that a name based on DNA alone could be shown to be “demonstrably ambiguous” (as required for epitypification under Art. 9.9) would be availability of longer sequences that contain the DNA marker used as type but have increased resolution in other portions that reveal cryptic species. In the FNS, we suggest that the Article on epitypification is pulled out from the proposal and voted on separately, after the main part of the proposal is considered.

Within the NCF there was low support for Prop. F-005 (2 – 11 – 1), with a 79% No vote. Within the ICTF, the Yes vote was 38% and the No vote was 54% (5 – 7 – 1).

### Section Y (new) - concerning genomic sequences as types

*Prop. F-006* [Thines, Cai, Wijayawardene, Phukhamsakda & Miller] Introduce a new Section Y in *Chapter F* “Genomic sequences as types”, with the following new articles, recommendations and notes

“*F.Y.1.* For organisms treated as fungi, on or after 1 January 2026, the holotype or epitype (Art. 9.1, 9.9, 9.21, 40.5) may also be an effectively published genomic sequence (see Art. F Y.4, F Y.5) if it is technically unfeasible to preserve a specimen or pure culture preserved in a metabolically inactive state that would show the features attributed to the taxon by the author of the name or if there are technical difficulties that prevent preservation of a specimen in a way suitable for later analyses.”

“*Note 1.* For the purposes of Art. F Y.1, preservation of a physical type for later use is technically unfeasible if there is no preservation method available that conserves diagnostic features or would allow for later nucleic acid extraction and sequence analyses with technologies available at the time of publication.”

“*F.Y.2.* For organisms treated as fungi, in order to be validly published as required by Art. 38.1, 38.2, 39.1, and 39.2 a name of a new taxon for which the type is a genomic sequence does not require a separate Latin or English diagnosis or description, or a reference to a previously and effectively published Latin or English description or diagnosis (see Art. 38.13). Instead of a description or diagnosis, a statement of why it is believed that the taxon is unnamed and an explanation of why a type specimen could not be isolated, cultured, or otherwise prepared must be provided.”

“*Note 2.* For the purposes of Art. 38.1, a genomic sequence designated as the type is itself treated as a description.

***Recommendation 1. If several related species are described based on a genomic sequence as the type, authors should add a diagnosis by listing diagnostic positions in a pairwise or multiple alignment with the appropriate coordinates (e.g. Kruse & al. in IMA Fungus 9(1): 49–73, Table 2, Fig. 6, 2018).”***

“*F.Y.3.* The genomic sequence type is to be deposited in a recognized repository (App. Y) and must not be changed (but see Art. F.Y.5) and the unique identifier issued by the repository is to be cited when a name is introduced based on that sequence. In order to effect typification, the citation of the identifier issued for the genomic sequence type by a recognized repository (App. Y) is sufficient.”

“*F.Y.4.* To be permissible as a type, a genomic sequence must belong to the nuclear genome of an organism treated as a fungus.”

“*F.Y.5.* A genomic sequence permissible as a type must be derived from a single sample, consist of 1 to 10,000 sequence parts (contigs) that collectively constitute the genomic sequence, and contain at least one continuous genomic sequence fragment larger than 200 kb. If later analyses establish that the genomic sequence type contains sequence data not belonging to the same species, nothospecies, or infraspecific taxon, the name remains typified by the largest genomic sequence fragment and all other sequence fragments unequivocally identifiable as belonging to the same taxon.”

“*Note 4*. A continuous sequence means a sequence without interspersed unidentified nucleotides (“Ns”) in case of assembled sequences. In case of single reads, the average read quality must exceed a Phred score of 20.”

“*F.Y.6.* The Nomenclature Committee for Fungi, in consultation with the General Committee, after seeking advice from relevant specialist committees and international societies as appropriate, appoints one or more open and accessible electronic repositories to issue the identifiers required by Art. F.Y.3 (see App. Z), and may cancel such appointments if the appropriate standards to issue identifiers in line with the requirements of Art F.Y.3 are not met. The Nomenclature Committee for Fungi has the responsibility to maintain a list of approved repositories.”

***Secretaries' comments*** Some aspects of this proposal mirror the wording of Prop. 005. Key differences are: (1) the requirement for the type to be an assembled nuclear genome rather than a DNA sequence, (2) treating the genome sequence itself as the description (but not requiring a description to be published), (3) recommending (rather than mandating) a diagnosis, (4) no provision for approved journals, and (5) epitypification with a genome only type allowed (for all names, not just those based on genomes as types).

In essence, the proposal allows for the straightforward description of a new species of fungus from an assembled nuclear genome where it is not feasible to preserve a specimen or pure culture stored metabolically inactive.

There is technical detail in the proposed Art F.Y.5, such as the minimum size for “at least one continuous genomic sequence fragment” and the read quality measure (“Phred score”). There are also technical specifications in the proposed Recommendation 1 which mentions “pairwise or multiple alignment”. If the Article is accepted, such technical terms will need to be defined in the Glossary.

In reference to the technical specifications, the proposal argues that “the introduction of a stability-promoting quality threshold is not alien to the ICNafp”. However, the examples of standards provided (such as “full and direct” citation of a basionym) are not comparable to the technical standards proposed (such as Phred scores and sequence length). The requirement for such standards is understandable but we consider that such technical detail does not belong in the *Code*, which concerns nomenclature not taxonomy. We note that for a description or diagnosis based on a conventional type there is no specification in the *Code* about the particular characters to be recorded, the methods by which these should be examined (such as in which mountants), the number of individuals to be measured, or the reference works to be utilized (such as specific colour charts).

As discussed in the Synopsis of proposals for the San Juan IMC (May & Redhead 2018), the responsibility for development of taxonomic standards for types that are genomic sequences could be placed with external bodies. Such a mechanism for determining standards would also deal with the issue that standards for genomes may well change as technology advances. There are examples of existing provisions of the *Code* that reference external bodies, such as the recognized repositories that issue identifiers for nomenclatural novelties of fungi (Art. F.5) and the “appropriate international bodies” that are involved in the setup of working groups that prepare lists for protection (Art. F.2.1). Another means of ensuring adherence to taxonomic standards is post-publication approval of names by a working group designated in the *Code*.

Within the NCF there was low support for Prop. F-006 (2 – 11 – 1), with a 79% No vote. In contrast, within the ICTF there was support for the proposal, with a 62% Yes vote (8 – 5 – 0).

## Procedures and further proposals

Procedures during the FNS are laid out in the *Shenzhen Code*. At the commencement of the Maastricht FNS, there will be a motion to accept the *Shenzhen Code* and the *San Juan Chapter F* the basis for discussion. An agenda covering the order of proceedings will be made available via the IMA website and included in the IMC on-line program. The Fungal Nomenclature Session will be scheduled in three blocks on Thursday 15 August. 10:30–12:30 (Nomenclature A), 12:45–13:45 (Nomenclature – Special session on DNA sequences as types) and 14:30–16:30 (Nomenclature B). The special session on DNA sequences as types is scheduled at that time to avoid a clash with any other symposia to facilitate voting by all those who wish to participate. The formal votes on Prop. F-005, F-006 and F-007 will be scheduled for the special session on DNA sequences as types.

Proposals not covered in this Synopsis may be introduced "from the floor" during the FNS, once proposed by a registered attendee of the FNS and seconded by five other registered attendees. It is important to note that during the five-day Nomenclature Section of an IBC, proposals "from the floor" have not been treated as merely "any other business" at the end of the Section, but traditionally have been introduced when important issues have not been addressed by the deadline for publishing proposals prior to the Congress, or when proposals accepted earlier in the week are realized to have unintended consequences that need rectifying. At the Shenzhen Congress, motions from the floor were ruled as having to be submitted by the penultimate day of the Nomenclature Section.

**We request that any further proposals that contain significant material are submitted to the Secretaries prior to the commencement of the IMC** (i.e. by Friday 9 August 2024), to allow time for such proposals to be made available to participants in the FNS. Any further proposals submitted by that time will be added to the IMA website <https://www.ima-mycology.org/index.php/formal-proposals>. We provide advance notice here that at the commencement of the FNS we will table a motion that new “proposals from the floor” must have been provided to the Secretaries before the commencement of the Session (in a digital format). Should that motion be approved, during the Session it will be permissible to move a motion from the floor to deal with material already on the agenda, but not to introduce new material.

During the Fungal Nomenclature Session, there will be time to debate the merits or otherwise of proposals and it is often the case that minor amendments are made during the course of such a debate. However, any significant changes to the proposals included in this Synopsis should be provided to the Secretaries well in advance of the FNS, to allow for due examination of consequences and interactions with other provisions.

## Opportunity to refine proposals concerning DNA sequences or genomes as types

We note that during the IMC there is a symposium sponsored by the ICTF on “DNA sequences as type equivalent - where to next?” to be held on Monday 12 August, 16:30–18:30. Given the complexity of Prop. F-005 and Prop. F-006, which both contain multiple paragraphs, this symposium provides an opportunity for mycologists to examine and discuss the proposals. If there are significant modifications to the proposals that would make them more likely to be supported, this is the time to finalise such modifications, as there will be limited time during the Fungal Nomenclature Session to make such modifications.

## Index of proposals

*F-001.* Enable the same epithet to be retained for different morphs of the same fungus. Insert a new provision in **Art.F.8**.

*F-002.* Clarify that a proposal to conserve a name with a conserved type does not require citation of a typification identifier. Amend **Art. F.5 Note 3**.

*F-003*. Remove the listing of synonyms from entries for protected names in the Appendices to the code and clarify the process of protection. Amend **Art. F.2.1**.

*F-004*. Clarify the processes of rejection. Amend Art. **F.7.1**.

*F-005*. Allow the naming of fungi from DNA sequences as types. Introduce a new **Section X** in *Chapter F* “DNA sequences as types”, with new Articles and Notes.

*F-006*. Allow genomic sequences to serve as types of names of organisms treated as fungi. Introduce a new **Section Y** in *Chapter F* for “Genomic sequences as types”, with new Articles and Notes.

*F-007*. Add a Recommendation on the designation of fungal organisms only known from DNA sequence data. Insert a new Recommendation and Example under **Art. F.5.5**.
